# Efficacy of Ketamine in Improving Pain after Tonsillectomy in Children: Meta-Analysis

**DOI:** 10.1371/journal.pone.0101259

**Published:** 2014-06-30

**Authors:** Hye Kyung Cho, Kyu Won Kim, Yeon Min Jeong, Ho Seok Lee, Yeon Ji Lee, Se Hwan Hwang

**Affiliations:** 1 Department of Pediatrics, Graduate School of Medicine, Gachon University, Incheon, Korea; 2 Department of Otolaryngology-Head and Neck Surgery, College of Medicine, The Catholic University of Korea, Seoul, Republic of Korea; Chiba University Center for Forensic Mental Health, Japan

## Abstract

**Background and objectives:**

The goal of this meta-analysis study was to perform a systematic review of the literature on the effects of ketamine on postoperative pain following tonsillectomy and adverse effects in children.

**Subjects and Methods:**

Two authors independently searched three databases (MEDLINE, SCOPUS, Cochrane) from their inception of article collection to February 2014. Studies that compared preoperative ketamine administration (ketamine groups) with no treatment (control group) or opioid administration (opioid group) where the outcomes of interest were postoperative pain intensity, rescue analgesic consumption, or adverse effects (sedation, nausea and vomiting, bad dream, worsening sleep pattern, and hallucination) 0–24 hours after leaving the operation room were included in the analysis.

**Results:**

The pain score reported by the physician during first 4 hours and need for analgesics during 24 hours postoperatively was significantly decreased in the ketamine group versus control group and was similar with the opioid group. In addition, there was no significant difference between ketamine and control groups for adverse effects during 24 hours postoperatively. In the subgroup analyses (systemic and local administration) regarding pain related measurements, peritonsillar infiltration of ketamine was more effective in reducing the postoperative pain severity and need for analgesics.

**Conclusion:**

Preoperative administration of ketamine systemically or locally could provide pain relief without side-effects in children undergoing tonsillectomy. However, considering the insufficient evaluation of efficacy of ketamine according to the administration methods and high heterogeneity in some parameters, further clinical trials with robust research methodology should be conducted to confirm the results of this study.

## Introduction

Tonsillectomy continues to be one of the most common otorhinolaryngology surgical procedures for children. Despite advancements in surgical and anesthetic techniques, severe pain and difficulty swallowing are common complaints encountered in children [1], [2]. Since pain relief after tonsillectomy can help patients recommence food intake and can prevent patient dehydration secondary to low food intake, various studies have been done to find effective methods of pain management with varying results. Many therapeutic modalities ranging from non-steroidal anti-inflammatory agents (NSAIDs), systemic opioids and local anesthetics have been used in children as effective means for post tonsillectomy pain control [3]. However, systemic opioids may cause respiratory depression, sedation, or nausea and vomiting. In contrast, NSAIDs cause less drowsiness, respiratory depression, and vomiting, but they may interfere with bleeding [4].

Ketamine is an intravenous anesthetic in phencyclidine family, which is a noncompetitive antagonist of N methyl-D-aspartate (NMDA) receptors. Ketamine influences the regulation of central sensitization and opium resistance [5]. Recent results of several studies in children using ketamine preemptively as an analgesic adjuvant have shown the effects of sub-analgesic doses of ketamine on postoperative pain and opioid consumption. However, others failed to spare morphine for ENT surgery [6]. Therefore, there is currently no sufficient evidence in the literature to fully support the use of ketamine in post-tonsillectomy pain in children. Considering that tonsillectomy continues to be a popular operation and post-tonsillectomy pain is the most common morbidity of tonsillectomy, it is important to ensure that clinicians follow effective practices for decreasing postoperative morbidity. This study assessed the evidence for the efficacy of ketamine for improving the patient experience of tonsillectomy.

## Materials and Methods

### Search strategy and selection of studies

Clinical studies published in English were identified from MEDLINE, SCOPUS, and the Cochrane Register of Controlled Trials up to the cutoff publishing date of February 2013. The following search terms were used: ‘tonsillectomy’, ‘adenotonsillectomy, ‘ketamine’, ‘pain’, ‘children’, ‘analgesics’, and ‘opioid’. Only studies published in English were selected for inclusion. Reference lists of identified studies were also checked to ensure that no relevant studies were missed.

**Figure 1:**
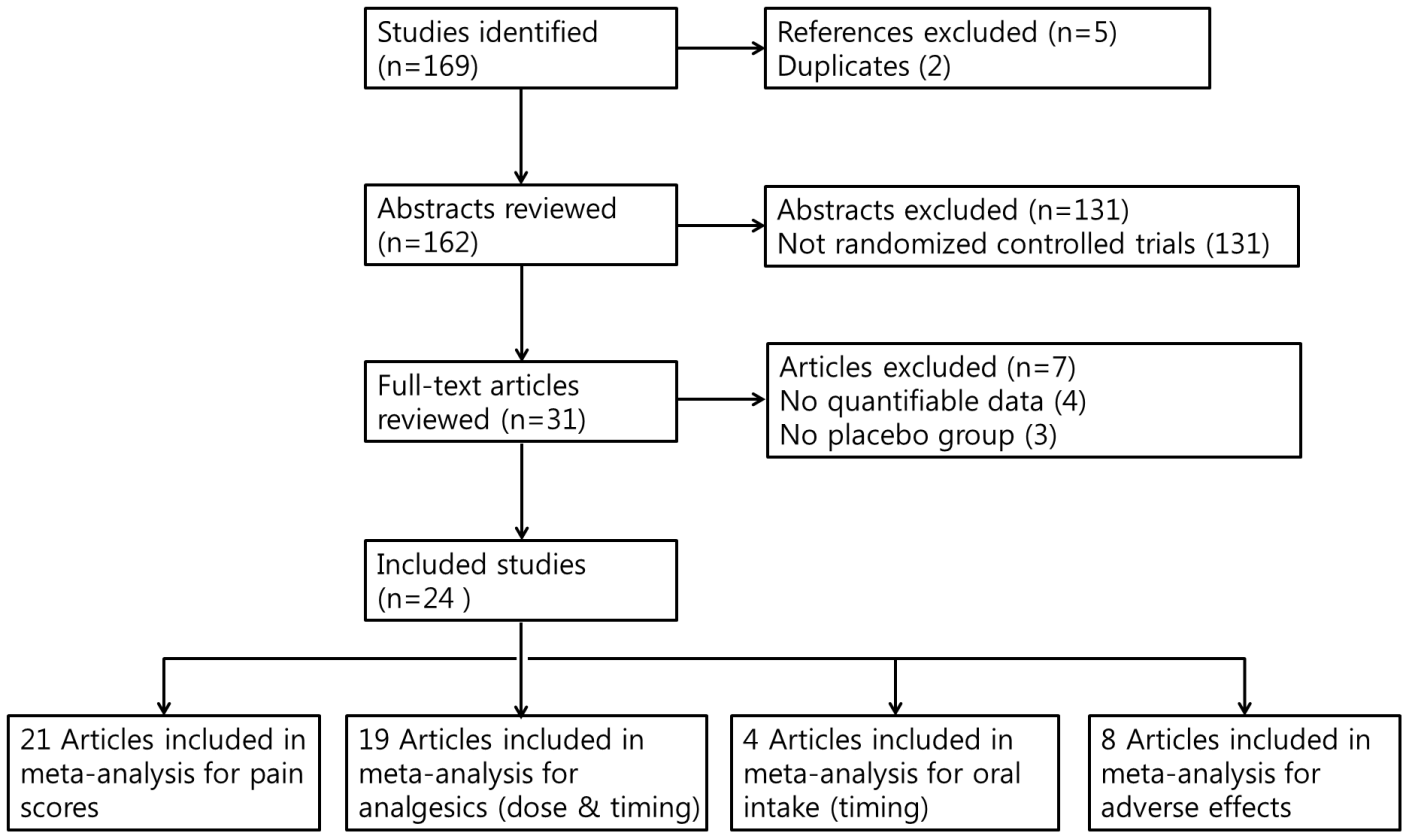
Diagram of selection of studies.

Two literature reviewers independently screened all abstracts and titles for candidate studies and discarded studies that were not related to the preoperative administration of ketamine. Full text of studies that were potentially relevant to the topic were obtained if a decision for inclusion could not be made from the abstract alone. Criteria for considering studies for this review were: randomized controlled trials of the analgesic effect of any route of ketamine administration on pain after tonsillectomy in children were included. Children (1–16 years of age) undergoing tonsillectomy (cold or thermal) for any indication as inpatient or day case. Studies with more than 10 patients per treatment group were included, which compared the primary outcomes were pain and comfort outcomes (visual analogue scale) compared topical anesthetic spray versus control (no treatment or opioid). Studies compared the analgesic effect on patients receiving the preoperative ketamine with those that used no treatment or opioid. Studies were excluded if, in addition to tonsillectomy, patients underwent additional procedures such as nasal and otologic surgery, or if reports were duplicated. In addition, studies were excluded from the analysis if clinical outcomes of interest were not clearly reported with quantifiable data, or it was not possible to extract and calculate the appropriate data from the published results. However, to include a maximum of appropriate studies, incomplete data were managed by contacting the corresponding author or estimation of the mean and the standard deviation on the basis of the sample size, median, and range according to the method described by Hozo and collaborator [7]. Figure 1 summarizes the search strategy used to identify studies selected for meta-analysis. Lists of excluded full text articles along with the reasons for their exclusion are provided in “File S1”.

### Data extraction and risk of bias assessment

Data from included studies were extracted using standardized forms and independently checked by the two reviewers. Outcomes analyzed were postoperative pain (pain scores after leaving the operation room) [2]–[5], [8]–[23], postoperative (24 hours) analgesic requirements (either doses or percentage of patient receiving postoperative opioids or nonopioids analgesics)[2]–[4], [8]–[10], [12]–[14], [16], [19], [20], [22]–[25], time to first analgesic administration (opioid or nonopioid analgesics administered with a defined pain intensity target)[3]–[5], [8]–[10], [12]–[14], [18], [22], [23], [26], time to first oral uptake [2], [5], [13], [14], postoperative nausea and vomiting (percentage of patients or dose of antiemetics) [2], [8], [9], [12]–[18], [24], [25] and the occurrence of psycho-mimetic manifestations including nightmares [9], [10], [18], sleep pattern change (percentage of patients) [9], [10], [12], [19], hallucinations (percentage of patients) [5], [8], [18], [19], [25] or sedation (sedation scores) [4], [5], [8], [9], [11], [15], [17], [24]. These outcomes were compared with preoperative ketamine group (including intravenous and peritonsillar administration) versus control group (no treatment) or opioid group (patients that used opioids) on postoperative period (0–24 hours after leaving the operation room).

The influence of the administrated ketamine on pain and sedation was separately analyzed through a grading scale after leaving the operation room. The evaluation was usually reported by the physician, with the smallest number being reported as no complaint or alert state, and the greatest number being reported as the severe complaint imaginable or asleep with no response to physical stimuli. From the studies marked for inclusion about the influence of administrated ketamine on postoperative pain and adverse effects, we abstracted data regarding patients number, grading scale used, amounts of analgesics intake, time to first oral intake or analgesics administration, incidences or percentage of adverse effect, and the *p*-value recorded in comparison between preoperative ketamine use [8], [9], [14]–[18]versus the control group or opioid group. In the event of missing or incomplete data, attempts were made to request further details of the published results from the authors directly.

The risk of bias for each study was evaluated using the Cochrane ‘Risk of Bias’ tool; the tool assesses the following types of biases: selection bias (random sequence generation and allocation concealment), performance bias (blinding of participants and personnel), detection bias (blinding of outcome assessment), attrition bias (incomplete outcome data), and reporting bias (selective reporting).

### Statistical analysis

The meta-analysis of selected studies was performed with ‘R’ statistical software (R Foundation for Statistical Computing, Vienna, Austria). When original data were expressed as continuous variables, meta-analysis was performed using the standardized mean difference (SMD). This method was chosen to calculate effect sizes due to the absence of a standardized scale used across all studies to assess postoperative pain (OPS, CHEOPS, and FACES score), postoperative analgesic requirements (amounts of postoperative opioid or nonopioid analgesics), time to first analgesic administration and first oral uptake, and postoperative sedation (sedation scores). In all other cases, outcome incidence analysis was performed using the odds ratio (OR) calculated using the Mantel–Haenszel method.

Heterogeneity was calculated with the I^2^ test, which describes the rate of variation across studies because of heterogeneity rather than probabilistic chance. The measure ranged from 0 (no heterogeneity) to 100 (maximum heterogeneity). All results were reported with a 95% confidence intervals (CI), and all *p*-values were two-tailed. When significant heterogeneity among outcomes was found (defined as I^2^>50), the random-effects model, according to DerSimonian-Laird, was used. This model assumes that the true treatment effects in individual studies may be different from one another, and that these are normally distributed. In addition, subgroup analysis was performed. Those outcomes that did not present a significant level of heterogeneity (I^2^<50) were analyzed with the fixed-effects model. The fixed-effects model uses the inverse variance approach, and it is assumed that all studies come from a common population. We used a funnel plot and Egger’s test simultaneously to detect potential publication bias and used the Duval and Tweedie’s trim and fill to adjust for missing studies and correct the overall effect size regarding publication bias.^15^ Additionally, sensitivity analyses were carried out in order to estimate the influence of each individual study in the meta-analysis results. This was performed by repeating the meta-analyses while omitting a different study each time.

## Results

A total of 24 studies with 1257 participants were included and reviewed for the meta-analysis. The results of bias assessment and study characteristics are described in Table 1.

**Table 1:** Summary of Studies Included in the Meta-analysis.

Study (year)	Sample Size	Comparison	Outcome Measure Analyzed	Judgment of Risk of Bias
Aspinall	50	Preoperative ketamine	Time to first analgesia (minutes)	low
(2001)		vs opioids (IV)		
Umuroğlu	60	Preoperative ketamine	Time to first analgesia (minutes)	low
(2004)		vs control or opioids (IV)	Incidence of analgesic requirements	
			Pain scores (CHEOPS)	
			Adverse effect (sedation score)	
Hasnain	80	Preoperative ketamine	Time to first analgesia (minutes)	low
(2012)		vs opioids (IV)	Incidence of analgesic requirements	
			Pain scores (CHEOPS)	
			Adverse effect (nausea and vomiting,	
			hallucination, sleep pattern, bad dream)	
Safavi	60	Preoperative ketamine	Postoperative analgesic requirements (mg)	low
(2012)		vs control (IV)	Postoperative antiemetic requirement (mg)	
Honarmand	60	Preoperative ketamine	Time to first analgesia (hours)	unclear risk
(2013)		vs control (IV)	The time to first oral intake (min)	
			Pain scores (CHEOPS)	
			Postoperative analgesic requirement (mg)	
			Adverse effect (sedation score, nausea and	
			vomiting)	
Taheri	60	Preoperative ketamine	Time to first analgesia (min)	low
(2011)		vs opioids (IV)	Incidence of analgesic requirements	
			Postoperative analgesic requirement (mg)	
			Pain scores (OPS)	
			Adverse effect (sedation score)	
Marcus	78	Preoperative ketamine	Time to first analgesia (min)	low
(2000)		vs opioids (IV)	Incidence of analgesic requirements	
			Adverse effect (sedation score, nausea and	
			vomiting, bad dream, sleep pattern)	
			Pain scores (CHEOPS)	
Ayatollahi	126	Preoperative ketamine	Pain scores (CHEOPS)	low
(2012)		vs control or opioids	Time to first analgesia (hours)	
		(topical)	The time to first oral intake	
			Adverse effect (sedation score,	
			hallucination)	
Tekelioglu	60	Preoperative ketamine	Pain score (Face score)	low
(2013)		vs control or opioids	Adverse effect (sedation score)	
		(topical)		
O’Flaherty	39	Preoperative ketamine	Pain score (CHEOPS)	unclear risk
(2003)		vs control (IV)	Postoperative analgesic requirement (mg)	
			Adverse effect (sedation scores,	
			hallucination, sleep pattern)	
Batra	40	Preoperative ketamine	Time to first analgesia (hours)	low
(2007)		vs control (IV)	Postoperative analgesic requirement (mg)	
			Pain score (CHEOPS)	
			Adverse effect (nausea and vomitting, sleep	
			pattern)	
Khademi	38	Preoperative ketamine	Incidence of analgesic requirements	low
(2010)		vs control (IV or topical)	Time to first analgesia (hours)	
			The time to first oral intake (hours)	
			Adverse effect (nausea and vomitting)	
			Pain score (Face score)	
Levänenn	40	Preoperative ketamine	Pain score (Face score)	low
(2000)		vs opioids (IV)	Postoperative analgesic requirement	
			Adverse effect (sedation score, nausea and	
			vomiting, sleep pattern)	
Elshamma	30	Preoperative ketamine	Pain score (VAS)	low
(2011)		vs opioids (IV)	Adverse effect (sedation score, nausea and	
			vomiting, sleep pattern)	
Erhan	60	Preoperative ketamine	Time to first analgesia (hours)	low
(2007)		vs control (topical)	Postoperative analgesic requirement (mg)	
			Pain score (CHEOPS)	
			Adverse effect (hallucination)	
Elhakim	50	Preoperative ketamine	Time to first analgesia (hours)	low
(2003)		vs control (IV)	Postoperative analgesic requirement (mg)	
			Pain score (CHEOPS)	
			The time to first oral intake (hours)	
			Adverse effect (nausea and vomiting,	
			hallucination, sleep pattern, time to ke)	
Ugur	75	Preoperative ketamine	Pain score (CHEOPS)	low
(2013)		vs control or opioids	Postoperative sedation scores	
		(topical)	Adverse effect (nausea and vomiting)	
Pirzadeh	60	Preoperative ketamine	Pain score (CHEOPS)	low
(2012)		vs control (topical)	Adverse effect (sedation scores, nausea	
			and vomiting)	
Dal	120	Preoperative ketamine	Incidence of analgesic requirements	low
(2007)		vs control (IV)	Time to first analgesia (hours)	
			Pain score (OPS)	
			Adverse effect (sedation scores, nausea	
			and vomiting, time to awake, bad dreams,	
			sleep pattern)	
Honarmandn	50	Preoperative ketamine	Pain score (CHEOPS)	unclear risk
(2008)		vs control (topical)	Time to first analgesia (hours)	
			Postoperative analgesic requirement (mg)	
			Adverse effect (nausea and vomiting,	
			hallucination)	
Canbay	45	Preoperative ketamine	Pain score (CHEOPS)	low
(2008)		vs control or opioids	Time to first analgesia (hours)	
		(topical)	Postoperative analgesic requirement (mg)	
			Adverse effect (sleep pattern, bad dreams)	
Eghbal	66	Preoperative ketamine	Pain score (Face score)	low
(2013)		vs control (IV)	Postoperative analgesic requirement (mg)	
			Adverse effect (nausea and vomiting)	
El Sonbaty	50	Preoperative ketamine	Pain score (OPS)	low
(2011)		vs opioids (topical)		
Siddiqui	50	Preoperative ketamine	Pain score (CHEOPS)	low
(2013)		vs control (topical)	Time to first analgesia (hours)	
			Adverse effect (nausea and vomitting, bad	
			dreams, hallucination)	

### Administration of ketamine versus control or opioid (pain)

**Figure 2:**
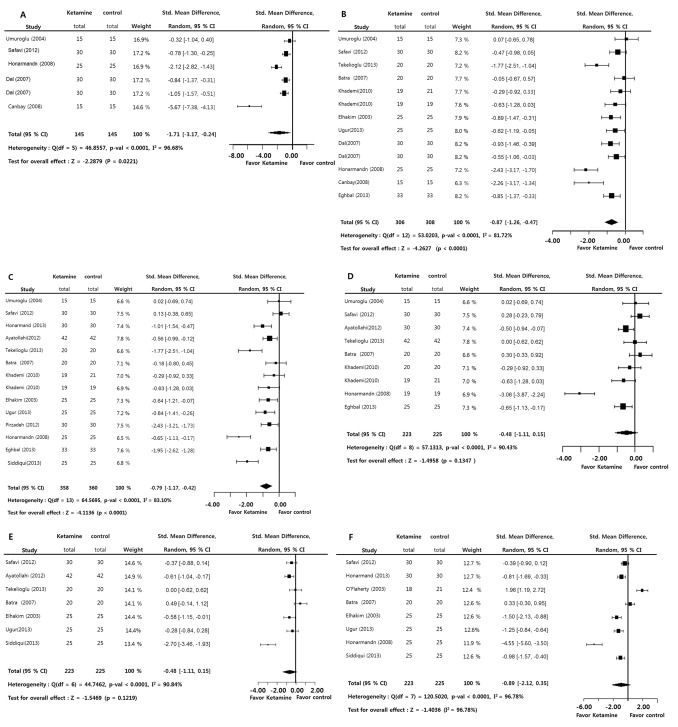
Preoperative ketamine versus control. Standard mean difference of pain at 0 (A), 1 (B), 4 (C), 6 (D), 12 (E), and 24 hours (F) from leaving operation room (total : number of participants per group).

Postoperative pain at 0 (SMD = −1.7085; *p* = 0.0221), 1 (SMD = −0.8660; *p*<0.0001), and 4 hours (SMD = −0.7945; *p*<0.0001) was statistically lower in the ketamine group versus the control group. However, there was no significant difference between both groups in the postoperative pain at 6 (SMD = −0.4813; *p* = 0.1347), 12 (SMD = −0.5600; *p* = 0.1219), and 24 hours (SMD = −0.8864; *p* = 0.1604). Significant inter-study heterogeneity was found at all periods (0∼24 hours; I^2^>50%). Egger’s test (*p<*0.05) at 0 and 1 hours showed some bias source in this sample of studies. However, Duval and Tweedie’s trim and fill at all postoperative periods showed no difference between observed and adjusted values. These results showed that the selective studies were not biased (Figure 2).

Results of the overall analysis did not take into account administration routes (intravenous vs peritonsillar). This could be reflected by the high heterogeneity (>80%) of the results obtained by all studies. Consequently, subgroup analyses were indicated. Analysis of ketamine efficacy according to the route of ketamine administration in postoperative periods showed that this factor could not influence analyzed outcomes (Table 2).

**Table 2:** Subgroup analysis of the effects of administration routes of ketamine on the postoperative pain.

	Effect size					
	0 hours	1 hours	4 hours	6 hours	12 hours	24 hours
**Overall results**	−**1.71**	−**0.87**	−**0.79**	−0.48	−0.48	−0.89
**(pain score)**	[−3.17, −0.24]	[−1.26, −0.47]	[−1.17, −0.42]	[−1.11, 0.15]	[−1.11, 0.15]	[−2.12, 0.35]
	**I^2^ = 96.68%**	**I^2^ = 81.72%**	**I^2^ = 83.10%**	I^2^ = 90.43%	I^2^ = 90.84%	I^2^ = 96.78%
	***p*** ** = 0.0221**	***p*** **<0.0001**	***p*** **<0.0001**	*p* = 0.1347	*p* = 0.1219	*p* = 1.0000
Intravenous administration	−**0.80**	−**0.52**	−**0.39**	−0.08	−0.17	−0.11
(pain score)	[−1.08, −0.52]	[−0.81, −0.23]	[−0.71, −0.08]	[−0.47, 0.31]	[−0.79, 0.45]	[−1.25, 1.03]
	**I^2^ = 0.00%**	**I^2^ = 42.75%**	**I^2^ = 52.97%**	I^2^ = 55.43%	I^2^ = 72.06%	I^2^ = 94.51%
	***p*** **<0.0001**	***p*** ** = 0.0004**	***p*** ** = 0.0146**	*p* = 0.6720	*p* = 0.5900	*p* = 0.8506
Peritonsillar administration	−**2.88**	−**1.34**	−**1.22**	−1.02	−0.87	−2.22
(pain score)	[−5.58, −0.18]	[−2.04, −0.64]	[−1.80, −0.65]	[−2.33, 0.29]	[−2.04, 0.29]	[−4.44, 0.01]
	**I^2^ = 97.04%**	**I^2^ = 85.00%**	**I^2^ = 84.54%**	I^2^ = 94.58%	I^2^ = 93.89%	I^2^ = 96.56%
	***p*** ** = 0.0369**	***p*** ** = 0.0002**	***p*** **<0.0001**	*p* = 0.1255	*p* = 0.1423	*p* = 0.0500

**Figure 3:**
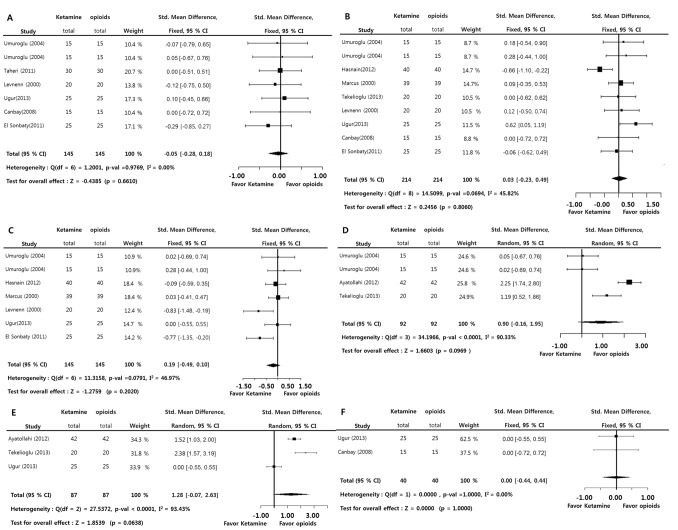
Preoperative ketamine versus opioids. Standard mean difference of pain at 0 (A), 1 (B), 4 (C), 6 (D), 12 (E), and 24 hours (F) from leaving operation room (total : number of participants per group).

There was no significant difference between both groups in the postoperative pain during all postoperative periods: 0 (SMD = −0.0516; *p* = 0.6610), 1 (SMD = 0.0330; *p* = 0.8060), 4 (SMD = −0.1912; *p* = 0.2020), 6 (SMD = 0.8959; *p* = 0.0969), 12 (SMD = 1.2775; *p* = 0.0638), and 24 hours (SMD = 0.0000; *p* = 1.0000). A significant inter-study heterogeneity was found at 6 hours (I^2^>50%). Egger’s test (*p<*0.05) at 1 hour indicated some bias source in this sample of studies. However, Duval and Tweedie’s trim and fill at all postoperative periods showed no difference between observed and adjusted values. These results showed that the selective studies were not biased (Figure 3).

### Administration of ketamine versus control (postoperative analgesic requirements, time to first analgesic administration, and time to first oral uptake)

**Figure 4:**
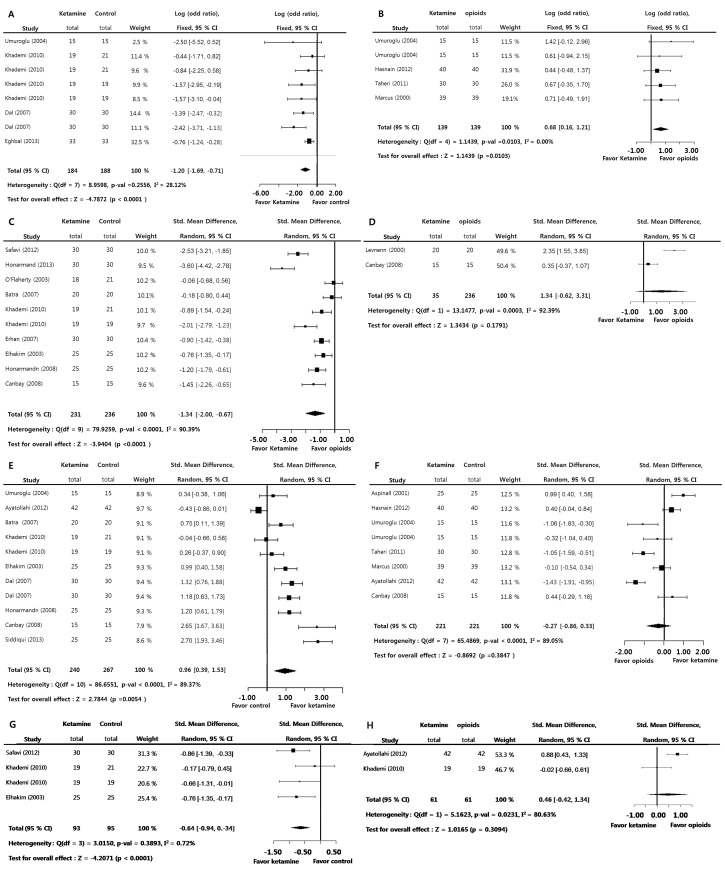
Preoperative ketamine versus control or opioids. Odd ratio of the incidence of analgesic requirements (A, B), standard mean difference of amounts of analgesic requirements (C, D), time to first analgesic administration (E, F), and time of first oral intake (G, H) (total : number of participants per group).

The incidence of analgesic requirements (LogOR = −1.1993; *p*<0.0001) and amounts of analgesic requirements (SMD = −1.3361; *p*<0.0001) during the 24-hour postoperative period was significantly lower in the ketamine group versus the control group. The time to first analgesic administration (SMD = 0.9601; *p* = 0.001) was significantly longer in the ketamine group versus the control group. However, the incidence of analgesic requirements on postoperative period (LogOR = −0.6850; *p* = 0.0103) was significantly lower in the ketamine group versus the opioid group, but there was no significant difference between the ketamine and opioid groups in the amounts of analgesic requirements during the postoperative period (SMD = 1.3442; *p* = 0.1791) and time to first analgesic administration (SMD = −0.2654; *p* = 0.3847). Significant inter-study heterogeneity was found on the amounts of analgesic requirements and time to first analgesic administration in comparison with control or opioids groups (I^2^>50%). Egger’s test (p<0.05) on the analgesic requirements and time to first analgesic administration in comparison with control groups indicated some bias source in this sample of studies. However, Duval and Tweedie’s trim and fill at the amounts of analgesic requirements and time to first analgesic administration showed no difference between observed and adjusted values. These results showed that the selective studies were not biased (Figure 4). In addition, regarding the issue of heterogeneity in the included studies, analysis of ketamine efficacy according to the routes of ketamine administration in postoperative periods showed that this factor could not influence analyzed outcomes (Table 3).

**Table 3:** Subgroup analysis of the effects of administration routes of ketamine on the consumption of analgesics.

	Effect size
**Overall results (Amount of analgesic control)**	−1.34 [−2.00, −0.67], I^2^ = 90.39%, p<0.0001
intravenous administration	−1.32 [−2.44, −0.20], I^2^ = 94.31%, p = 0.0210
Peritonsillar administration	−1.32 [−2.78, −1.23], I^2^ = 47.48%, p<0.0001
**Overall results (Time to analagesic uptake (control))**	0.96 [0.39, 1.53], I^2^ = 89.37%, p = 0.0054
intravenous administration	0.69 [0.21–1.17], I^2^ = 66.33%, P = 0.0048
Peritonsillar administration	1.22 [0.23–2.22], I^2^ = 93.59%, P = 0.0160
**Overall results (Time to analagesic uptake (opioids))**	−0.27 [−0.86, 0.33], I^2^ = 89.05%, p = 0.3847
intravenous administration	−0.17 [−0.81, 0.47], I^2^ = 87.40%, p = 0.6005
Peritonsillar administration	−0.51 [−2.34, 1.31], I^2^ = 94.37%, p = 0.5809

The time to first oral uptake (SMD = −0.6382; *p*<0.0001) was statistically shorter in the ketamine group versus the control group. No significant inter-study heterogeneity (I^2^<50%) and Egger’s test (*p*>0.05) on this measurement suggested that a bias source was not evident in this sample of studies. There was no statistically significant difference between the ketamine group and opioid group in this measurements (SMD = 0.4573; *p* = 0.3094). Although significant inter-study heterogeneity (I^2^>50%) was found in this measurement, Egger’s test was not measured because of the small sample size enrolled in the studies (Figure 4).

### Administration of ketamine versus control (adverse effect)

**Figure 5:**
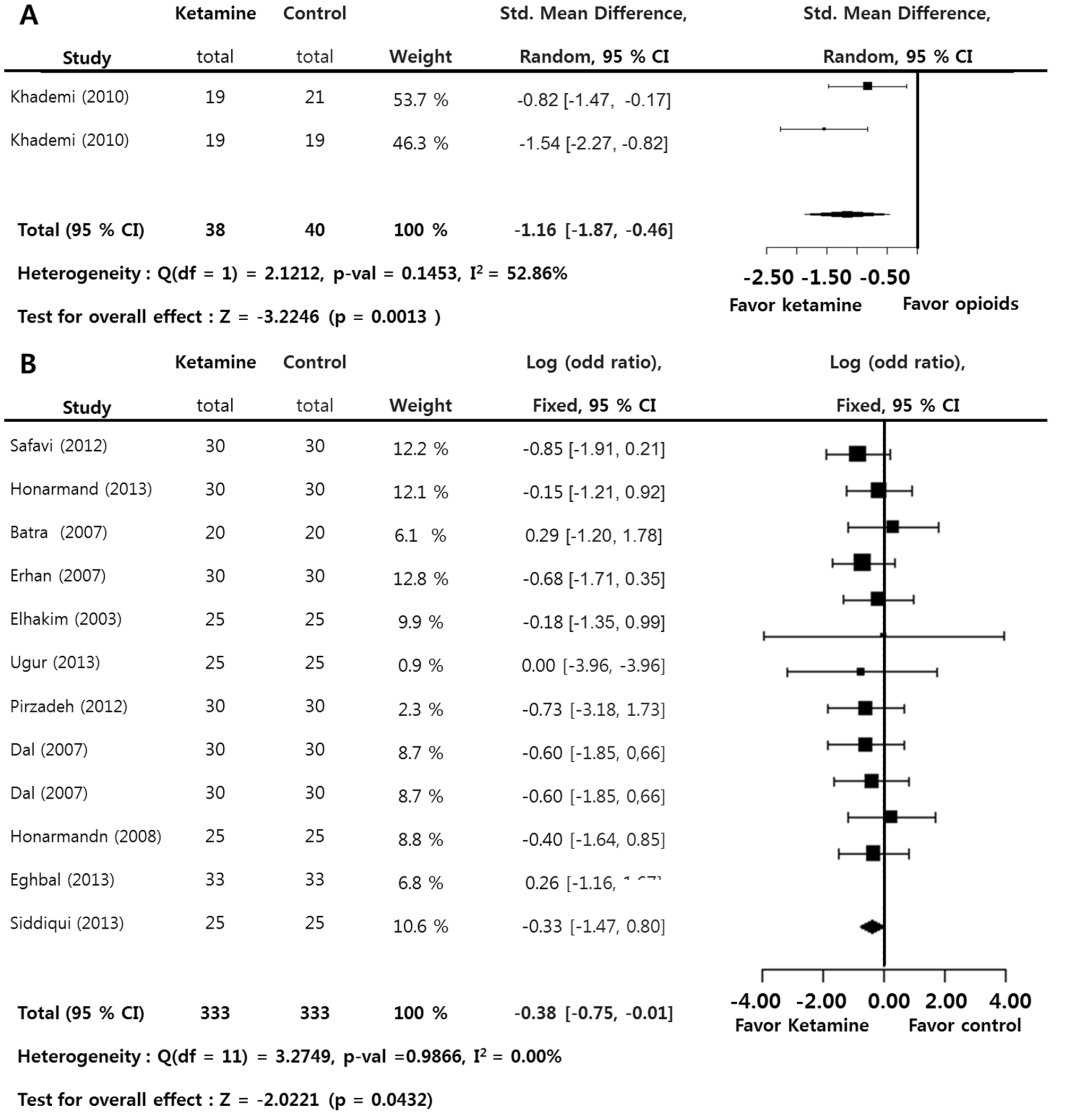
Preoperative ketamine versus control. Standard mean difference of amounts of antiemesis requirements (A) and odd ratio of the incidence of postoperative nausea and vomitting (B) (total : number of participants per group).

The incidence of postoperative nausea and vomiting (LogOR = 0.3253; *p* = 0.0432) and amounts of antiemetics requirements (SMD = −1.1611; *p* = 0.0013) during the 24-hour postoperative period was significantly lower in the ketamine group versus the control group. A significant inter-study heterogeneity approximately was not found on these measurements (I^2^<50%). Egger’s test (*p*>0.05) on these measurements suggested that a bias source was not evident in this sample of studies (Figure 5).

**Figure 6:**
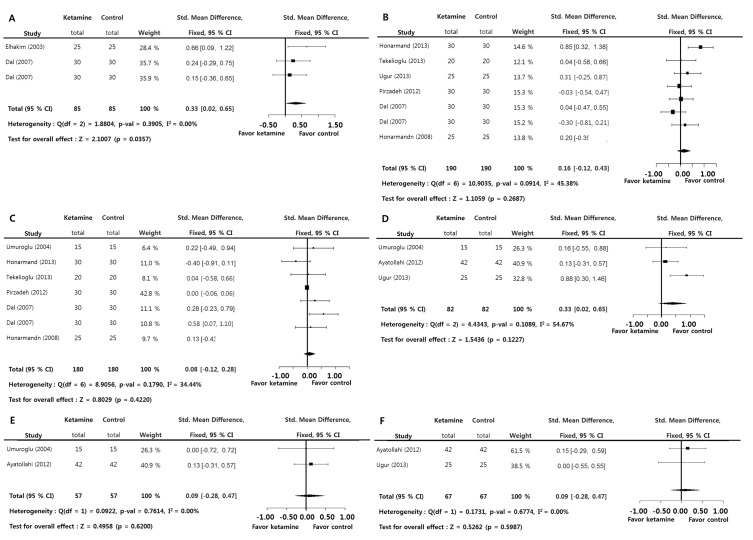
Preoperative ketamine versus control. Standard mean difference of the time to awake (A) and standard mean difference of the degree of sedation at 0 (B), 1 (C), 4 (D), 6 (E), and 12 (F) from leaving operation room (total : number of participants per group).

Although the time to awake (post anesthesia recovery; SMD = 0.3253; *p* = 0.0357) was significantly longer in the ketamine group versus the control group, the degree of sedation during the 12-hour postoperative period (0 (SMD = 0.1553; *p* = 0.2687), 1 (SMD = 0.0801; *p* = 0.4220), 4 (SMD = 0.3842; *p* = 0.1227), 6 (SMD = 0.0945; *p* = 0.6200), and 12 hours (SMD = 0.0923; *p* = 0.5987)) was not significantly larger in the ketamine group versus the control group. A significant inter-study heterogeneity approximately was not found on these measurements (I^2^<50%). Egger’s test (*p*>0.05) on these measurements suggested that a bias source was not evident in this sample of studies (Figure 6).

**Figure 7:**
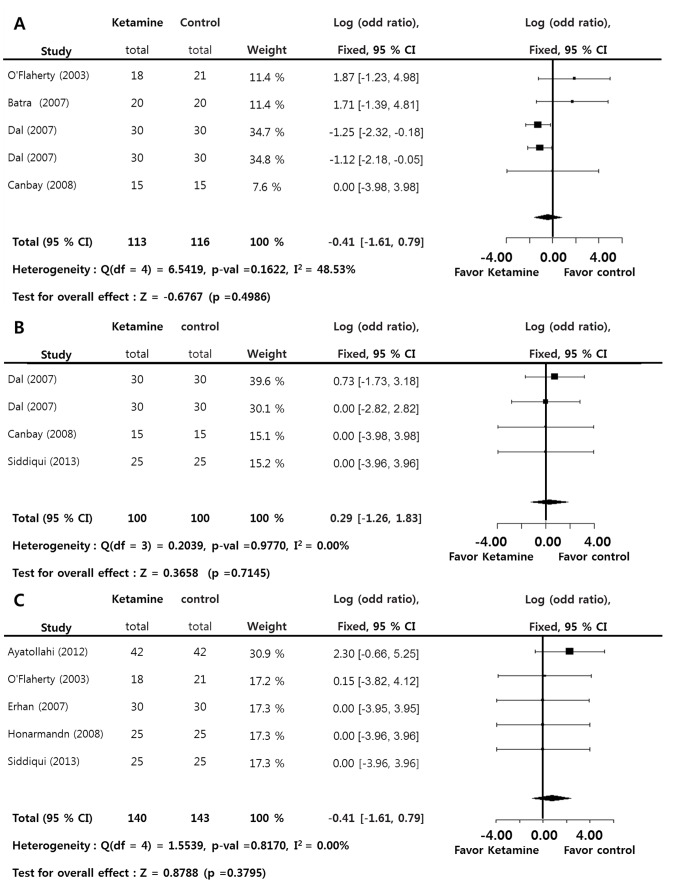
Preoperative ketamine versus control. Odd ratio of the incidence of worse sleep pattern change (A), bad dream (B), and hallucination (C) (total : number of participants per group).

Regarding the adverse psycho-mimetic effect, the incidence of worse sleep pattern change (SMD = −0.4144; *p* = 0.4986), bad dreams (SMD = 0.2885; *p* = 0.3658), and hallucination (SMD = 0.7374; *p* = 0.3795) showed no significant difference in the ketamine group versus the control group. Significant inter-study heterogeneity approximately was not found on these measurements (I^2^<50%). Egger’s test (*p*<0.05) on sleep pattern change in comparison with control groups suggested that some bias source was evidenced in this sample of studies. However, Duval and Tweedie’s trim and fill at these measurements showed there was no difference between observed and adjusted values. Therefore, these results showed that the selective studies were not biased (Figure 7).

### Sensitivity analyses

Sensitivity analyses were performed to evaluate whether the pooled estimates of postoperative pain, postoperative analgesic requirements, time to first analgesic administration, time to first oral uptake, and adverse effect were different by omitting a different study each time and repeating the meta-analyses. Finally, the results were all consistent with the above outcomes.

## Discussion

Post-tonsillectomy pain is one of the universal complaints of the patients and remains a considerable clinical problem. In particular, the intense pain itself can have an adverse effect both on the heart rate and blood pressure of the patient. The pain which the body senses acting via the sympathetic autonomous nervous system may increase the heart rate, and blood pressure [15]. Both of these effects causes a high cardiac output, which leads to postoperative exhaustion of the patient [27]. Therefore, pain assessment and control after tonsillectomy must be included in the routine post-tonsillectomy management of the patients.

The choice of the pain scoring system in children has great importance because the assessment of pain in children is difficult as their cognitive and communicative skills to report pain are underdeveloped. In previous studies [4], [28], several scoring systems (OPS, CHEOPS, and pediatric behavioral FLACC pain scale as three examples) were used to assess pain by the clinicians to obtain more reliable results. These systems depend on the behavioural expression and/or verbal expression, and are sensitive for this population and validated measures of pain for children [4]. In addition, the frequency or amount of analgesics used following tonsillectomy used to be adopted as an objective criterion for assessment of pain [29]. This study attempted to assess the extent of pain via the observational pain scale as well as the frequency or amount of analgesics used.

There are several systematic reviews related to pediatric tonsillectomy. First one by Hamunen el al. [30] examining opioids, NSAIDS and paracetamol for reducing pain following tonsillectomy and the others by Laskowski et al. [31] and Dahmani et al. [32] on ketamine for improving pain after tonsillectomy in children. Hamunen el al. reported that no analgesic in single prophylactic dose was enough to provide analgesia during the day of operation thus repeated administration was needed to guarantee freedom from pain. Combination of regularly administered mild analgesics and individually titrated opioids were needed to reach optimal result. However, they had major limitations that there were no review on adverse effects following analgesics and no data to show which analgesic and dose from each class of analgesics to choose.

There have been a number of investigations into the efficacy of ketamine in reducing postoperative pain, along with different routes (systemically or locally) with their various doses and schedules. Laskowski et al. suggested that intravenous ketamine is effective in reducing total opioid requirements and delaying the time to first analgesic dose in painful procedures, including upper abdominal, thoracic, and major orthopedic surgeries, while no significant benefit was found in those patients undergoing tonsillectomy. However, there were only four studies regarding pediatric tonsillectomy in all enrolled 47 studies [31]. Therefore, their results could not to reach a stronger conclusion regarding the efficacy of ketamine on post-tonsillectomy pain in children. By contrast, Dahmani et al. conducted to pool data from many studies regarding pediatric tonsillectomy and reached more stronger conclusion regarding the efficacy of ketamine on tonsillectomy in children [32]. However, there were several problems with the analysis used in this review. Dahmani et al. [32] performed an analysis of pain scores via the division of postoperative periods (0–24 hours) into two simple categories - postoperative care unit (PACU) stay (the first 2 hours) and the early postoperative period (between 6 and 24 hours) defined based on individual study results. However, because most of studies measured these values every approximately 2–3 hours, even 15 or 30 minutes, several time-dependent values could be included per single category. Therefore, the selection of one from several measured values without criteria of selection may have cause selection bias that could not reflect the typical values in the category. Additionally, the most annoying limitations of ketamine are its adverse psycho-mimetic effects, including dizziness, depersonalization, frightening dreams and hallucinations, although these are not common in low doses [3]. A previous study performed a meta-analysis of the efficacy of ketamine on postoperative pain without adverse effects [32].

In this study, we used more detailed time category to maximize the data included in the meta-analysis and to more precisely evaluate the time dependent change of the effect of ketamine on postoperative pain. We also assessed adverse effects for a comparison between ketamine and control groups. Additionally, ketamine at subanesthetic doses can prevent central hypersensitivity caused by pain [2]. Most of the studies evaluated the efficacy of preoperative systemic or local administration on the pain control after tonsillectomy. Therefore, this study included studies with narrow inclusion criteria (preoperative systemic or local administration methods and low dosage prior to the tonsillectomy).

Our results showed that the postoperative pain in the early postoperative period (0–4 hours) and frequency and amounts of analgesic intake during 0–24 hours postoperatively were statistically decreased in the ketamine group compared with the control group and the time to first analgesic administration was significantly longer in the ketamine group than the control group. Moreover, the SMD for the measurements regarding the pain and analgesics intake usually exceeded 0.8, which meant that these effect sizes were clinically significant during postoperative periods [33]. Considering the amounts of analgesics intake used as the objective criterion for the assessment of pain and pain scales reported by clinicians are reliable measure of pain for children according to age group, preoperative administration of ketamine after tonsillectomy considerably influenced postoperative pain and could reduce analgesic requirements.

The opioids injections are most commonly used to manage most patients after tonsillectomy, based on the simplicity and practical applicability of the technique [3], [25]. However, many children who underwent tonsillectomy to treat the obstructive sleep apnea may be sensitive to opioids, causing significant respiratory problems. It has been recommended that opioids be used with extreme caution and only short acting ones should be used if necessary [23]. Therefore, we also compared the analgesic effect of ketamine and opioids after tonsillectomy among children. There was no statistically significant difference between postoperative pain and frequency and amounts of analgesics intake during the 24-hour postoperative period. Considering that ketamine has less effect on airway patency and respiratory drive, these results indicated that ketamine might be an ideal choice in children with sleep apnea.

The incidence of adverse effects, such as sedation, sleep pattern change, dreaming, and hallucination, has limited the use of ketamine in anesthetic dose [23]. In this meta-analysis, the incidence of bad dreams, sleep pattern change or hallucinations in the ketamine group was low, and the degree of sedation as well as incidences of adverse events were similar with the control group, in accordance with previous data when low dose ketamine was used during anesthesia [3]. These results could suggest that the effects of low ketamine are relatively unimportant in the adverse effects. By contrast, there was low incidence of postoperative nausea and vomiting and decreased antiemetic prescriptions in the ketamine group in comparison to the control group. Previous studies have indicated that vomiting, which is induced by swallowed blood and oropharyngeal irritation, occurs in 40–65% of children after tonsillectomy. The administration of opioids has an additive effect on the incidence of nausea and vomiting [4] but ketamine has no additional emetogenic effect [12]. These facts could explain the positive effect of ketamine on emesis because of effective pain control and reduced analgesic requirements, including opioids.

The adverse effects of ketamine include increased production of salivary and tracheobronchial secretions as well as nausea, vomiting, and psycho-mimetic manifestations. In particular, increased secretions and stimulation of the posterior pharynx by suction instruments could cause the laryngospasm which would be considered to the most feared complication of ketamine administration, although this event is rarely observed [34]. Therefore anti-sialagogues such as atropine and glycopyrrolate had been recommended as a routine adjunct [35]. However, all episodes of laryngospasm essentially always have been transient and responded quickly to assisted ventilation and oxygen. In large case series of Epstein et al., no difficulty occurred in 1100 children who received ketamine without atropine [34]. Recently, large meta-analysis demonstrated that ketamine-associated transient laryngospasm and hypersalivation were relatively uncommon (0.3% incidence in children) and rare in anesthetic dose. Therefore, Green et al. suggested that routine prophylactic anti-sialagogues no longer recommended because of doubtful clinical importance [36]. In addition, low-dose ketamine can reduce its side-effects such as salivary and bronchial secretions [37]. Because of these facts, we could not find a controlled study of pediatric tonsillectomy assessing airway related problems, including laryngospasm or hypersalivation, when administered with ketamine in the subanaesthetic doses discussed here and could not perform the review on the ketamine-caused respiratory adverse effects via pooled data.

Subgroup analyses (systemic and local administration) regarding pain scores was applied to decrease the heterogeneity and to identify factors influencing the results. Systemic and local administrations of ketamine were effective in postoperative pain control. Preoperative ketamine administration through the intravenous and peritonsillar infiltration both reduce the incidence and severity of postoperative pain as well as the need for analgesics in children undergoing tonsillectomy. However, peritonsillar infiltration was more effective in reducing the pain severity and need for analgesics [13]. In particular, despite being statistically insignificant, peritonsillar infiltration of ketamine showed a large effect size (>0.8) during the late postoperative period (6–24 hours). These results were somewhat different from the results of Dahmani et al. [32] in that locally administered ketamine during tonsillectomy decreased both PACU (0–2 hours) and early (6–24 hours) postoperative pain. This discrepancy may reflect that our study used more detailed time categories and included more studies than the previous study [32].

Despite subgroup analyses, it was not possible to decrease the heterogeneity for the local administration of ketamine. Additionally local administration showed different effect from systemic administration in this study. There would be three reasons. Firstly, ketamine has peripheral and central effects. During peritonsillar infiltration, ketamine exerts local analgesic effect by blockade of sodium and potassium channels in the peripheral nerve (tonsillar nerve) as well as an anti-hyperalgesic effect. Secondly, the local anaesthetic effect of ketamine is more than 90 minutes and lasts for 1 week after infiltration in comparison with systemic pharmacokinetic with maximum plasma concentrations occurring 20–30 minutes after a dose of ketamine (0.5 mg/kg) and elimination half-life of 155 minutes [18], [28]. Thirdly, one factor that might have caused the high heterogeneity was the variability of the peripheral nerve block by different clinicians [32].

Although the results of this study offer evidence for the use of preoperative ketamine at low dose in ameliorating patient’s morbidity, the efficacy of ketamine when coadministered with opioid agents or when administered continuously during tonsillectomy remains unclear, as a meta-analysis comparing these particular factors has yet to be conducted. Additionally, assessing and reporting on the effects of other agents would also be useful to understand their potential impact on morbidity associated with tonsillectomy.

## Conclusion

This meta-analysis demonstrates that local or systemic administration of ketamine before tonsillectomy in children can decrease post tonsillectomy pain efficiently without adverse effect such as nausea and vomiting, sedation, bad dreams, sleep pattern change or hallucinations. It can also decrease analgesic consumption and the time to the beginning of oral liquid diet. In particular local administration of ketamine was more effective in reducing the postoperative pain severity and need for analgesics.

## Supporting Information

Checklist S1 PRISMA Checklist.
(DOC)

File S1 The list of articles excluded from this study with the reasons.
(DOC)
