# Supplementary material for: Efficacy of Ketamine in Improving Pain after Tonsillectomy in Children: Meta-Analysis
Source: PLoS One. 2014 Jun 30;9(6):e101259. doi: 10.1371/journal.pone.0101259 (PMC4076328; doi:10.1371/journal.pone.0101259)
Supplement: File S1 — The list of articles excluded from this study with the reasons. (DOC) [file pone.0101259.s002.doc]

**Articles excluded from this study**

**No quantifiable data (N=4)**

Anesth Pain Med. 2012;2(1):17-21. Comparison between effect of lidocaine, morphine and ketamine spray on post-tonsillectomypain in children. Hosseini Jahromi SA, Hosseini Valami SM, Hatamian S.

Korean J Anesthesiol. 2010;58(5):440-5. The effect of ketamine on the incidence of emergence agitation in children undergoingtonsillectomy and adenoidectomy under sevoflurane general anesthesia. Lee YS, Kim WY, Choi JH, Son JH, Kim JH, Park YC.

Paediatr Anaesth. 2006;16(9):962-7. Effect of an intravenous single dose of ketamine on postoperative pain in tonsillectomy patients. DA Conceição MJ, Bruggemann DA Conceição D, Carneiro Leão C.

S Afr Med J. 1987;72(12):839-42. Prevention of post-tonsillectomy pain with analgesic doses of ketamine. Murray WB, Yankelowitz SM, le Roux M, Bester HF.

**No placebo group (N=3)**

Clin J Pain. 2008;24(5):395-8. Ketamine does not reduce postoperative morphine consumption after tonsillectomy in children. Abu-Shahwan I.

J Clin Anesth. 2007;19(2):115-9. Pain prevention with intraoperative ketamine in outpatient children undergoing tonsillectomy or tonsillectomy and adenotomy. Aydin ON, Ugur B, Ozgun S, Eyigör H, Copcu O

Med Sci Monit. 2009;15(10):CR539-543. Intravenous ketamine and local bupivacaine infiltration are effective as part of a multimodal regime for reducing post-tonsillectomy pain. Inanoglu K1, Ozbakis Akkurt BC, Turhanoglu S, Okuyucu S, Akoglu E.

**Same dataset in different publications (n=2)**

The efficacy of preincisional peritonsillar infiltration of ketamine for postoperative analgesia in children following tonsillectomy Raees U.S., Siddiqui S.Z., Raza S.A., Siddiqui A.S. Anaesthesia, Pain and Intensive Care 2012;16(1):98-99

Preemptive peritonsillar ketamine infiltration: Postoperative analgesic efficacy versus meperidine ElSonbati M., Aboeldahab H., Mostafa A., Aboshanab O. Egypt J Anaesth 2009; 25(2):97-106
